# Reliability of Repeated Trials Protocols for Body Composition Assessment by Air Displacement Plethysmography

**DOI:** 10.3390/ijerph182010693

**Published:** 2021-10-12

**Authors:** Paul Muntean, Monica Micloș-Balica, Anca Popa, Adrian Neagu, Monica Neagu

**Affiliations:** 1 Department of Functional Sciences, Victor Babes University of Medicine and Pharmacy Timisoara, 300041 Timisoara, Romania; munteanpaul94@gmail.com (P.M.); moni.0912@yahoo.com (M.M.-B.); anca.lp@gmx.com (A.P.); neagu@umft.ro (A.N.); 2 Center for Modeling Biological Systems and Data Analysis, Victor Babes University of Medicine and Pharmacy Timisoara, 300041 Timisoara, Romania; 3 Department of Rehabilitation, Physical Medicine and Rheumatology, Victor Babes University of Medicine and Pharmacy Timisoara, 300041 Timisoara, Romania; 4 Department of Physics and Astronomy, University of Missouri, Columbia, MO 65211, USA

**Keywords:** BOD POD, precision, Bland–Altman analysis, technical error of measurement, standard error of measurement, minimal detectable change, intraclass correlation coefficient

## Abstract

Air displacement plethysmography (ADP) is fast, accurate, and reliable. Nevertheless, in about 3% of the cases, standard ADP tests provide rogue results. To spot these outliers and improve precision, repeated trials protocols have been devised, but few works have addressed their reliability. This study was conducted to evaluate the test–retest reliabilities of two known protocols and a new one, proposed here. Ninety-two healthy adults (46 men and 46 women) completed six consecutive ADP tests. To evaluate the reliability of single measurements, we used the results of the first two tests; for multiple measures protocols, we computed the test result from trials 1–3 and the retest result from trials 4–6. Bland–Altman analysis revealed that the bias and the width of the 95% interval of agreement were smaller for multiple trials than for single ones. For percent body fat (%BF)/fat-free mass, the technical error of measurement was 1% BF/0.68 kg for single trials and 0.62% BF/0.46 kg for the new protocol of multiple trials, which proved to be the most reliable. The minimal detectable change (MDC) was 2.77% BF/1.87 kg for single trials and 1.72% BF/1.26 kg for the new protocol.

## 1. Introduction

Body composition assessments are essential in sports medicine, for the optimization of physical performance [1], in body mass management, and for fighting the adverse effects of overweight and obesity [2], as well as in geriatric care, for tracking the age-related loss of fat-free mass [3]. Periodic evaluations of body composition are useful for elite athletes from sports in which excess fat mass hampers performance. Runners, for example, periodize their training and body composition because low body fat maintained for a long time might affect their health. Monitoring body composition and the intake of essential trace elements are recommended for the optimization of the training regimen of elite runners [4]. To track body composition variables is also important for the general population, to motivate people to adopt a healthy lifestyle [5], and for patients who suffer from medical complications of overweight and obesity [2]. Therefore, body composition studies are important for improving public health.

Air displacement plethysmography (ADP) is a noninvasive technique of body composition analysis by full body densitometry [6,7]. It does not expose the subject to ionizing radiations or to other harmful physical factors, so it is suitable for frequent assessments of the amount of fat present in the subject’s body [8]. ADP was used in combination with magnetic resonance imaging for the characterization of functional body composition-derived human phenotypes [9]. The strengths and limitations of various methods of body composition assessment have attracted much attention recently [10,11]. The only commercially available ADP instrument, the BOD POD^®^ (COSMED, USA), is used in clinical, commercial, and research settings because it is accurate, reliable, and subject-friendly [8].

Despite the carefully designed measurement process, individual BOD POD tests occasionally can lead to rogue results (outliers) [12,13,14,15,16]. They were spotted, in about 3% of the subjects [14], in reliability studies based on duplicate trials. By definition, an outlier is observed when the difference between two successive test results exceeds a certain threshold, of about twice the technical error of measurement of the instrument [14]. The cause of outliers is as yet unknown, but it has been argued that, whatever the disturbing factor is (such as a condition of the subject, the environment, or the instrument), it should last for several minutes in order to affect all the BV determinations involved in one test procedure [14]. Wells and Fuller demonstrated that differences between successive procedures arise almost exclusively due to biological factors [15]. Hence, these authors conjectured that rogue values might originate from unusual breathing and/or movement patterns associated with the subject feeling uncomfortable in the measurement chamber. They advised to conduct pairs of ADP procedures meant to identify and eliminate outliers [15].

Collins and McCarthy noticed that the first ADP procedure is an unknown experience for most subjects, potentially leading to an erroneous BV measurement [12]. Therefore, they proposed to perform at least two complete tests, followed by a third one if the difference in %BF between the first two tests is greater than 0.5%. The importance of multiple trials has also been emphasized by Tucker et al. [17], who proposed a repeated trials protocol similar to that of Collins and McCarthy, but with an acceptable difference of 1% BF between the first two tests. Assessing the reliability of the BOD POD in a sample of 283 middle-aged women, Tucker et al. computed the absolute mean difference in body fat percentage between pairs of trials, obtaining 0.96% BF. In contrast, comparing the closest two tests of the three conducted (when the difference between the first two exceeded 1% BF), the absolute mean difference decreased to 0.55% BF [17]. Moreover, the intraclass correlation coefficient was 0.991 for the first two values and 0.998 for the closest pair of values. Thus, the results reported by Tucker et al. suggest that a third trial (when necessary) can improve the test–retest reliability of the BOD POD.

Nevertheless, to our knowledge, the reliabilities of the repeated measures protocols developed to date have not been evaluated, yet. To do so, one needs (i) to conduct the given protocol at least twice, thereby obtaining the test and retest results, and (ii) to compute statistical measures of reliability [18,19].

The objective of this study was to evaluate the test–retest reliabilities of body composition assessments via the protocols of References [12,17], as well as the newly proposed “median” protocol, which consists in conducting triplicate measurements and taking the median of the three results. The hypothesis underlying this study was that protocols involving multiple measurements assure a better precision than single tests.

## 2. Materials and Methods

The present study was conducted in accordance with the ethical principles for medical research stated in the Declaration of Helsinki, and has been approved by the Committee of Research Ethics of our institution (protocol 20/24 July 2019). Prior to body composition testing, written informed consent was obtained from each subject.

### 2.1. Subjects

Study participants were recruited from the local community through social media and flyers. A total of 92 clinically healthy adults (46 men and 46 women) were included in this study. Table 1 presents the descriptive statistics of the study sample. The standard deviation (SD) of the BMI values is relatively large (19% of the mean BMI), indicating that our study sample was highly heterogeneous. Therefore, it enabled a comparison of the precision of measurement protocols over a wide range of body composition variables.

### 2.2. ADP Measurements

The BOD POD Gold Standard Body Composition Tracking System (COSMED USA, Concord, CA, USA) was used with software version 5.3.2. System quality check and scale calibration were carried out on a daily basis.

Participants were asked to refrain from eating or drinking for at least 4 h prior to the test. Upon their arrival to the lab, they were asked to use the restroom. Subjects were also instructed to remove jewelry and glasses, and to wear form-fitting clothing: either a Lycra^®^/spandex-type swim suit or single-layer compression shorts and a single-layer jog bra (without padding) for women. Their hair was thoroughly compressed by a Lycra swim cap, and special care was taken to eliminate air pockets trapped between hairs. The swim cap was put on before the first weighing and kept in the same position during the entire sequence of measurements, thereby avoiding variations in the volume of air maintained under isothermal conditions in the proximity of the scalp.

First, stature was recorded to the nearest 0.5 cm using a wall-mounted tape measure (GIMA 27335, GIMA, Gessate, Italy). The subject was instructed to maintain a horizontal orientation of her/his Frankfort plane while three height measurements were taken, and their median was recorded in the BOD POD’s software. Thoracic gas volume was predicted by the BOD POD’s software based on age, sex, and height [20].

We conducted 6 ADP trials in a row, with a total duration of 40–60 min. Each trial comprised (i) one body mass measurement to the nearest 0.001 kg, using the BOD POD’s scale, (ii) one volume calibration using the cylinder provided by the instrument’s manufacturer, and (iii) two assessments of the subject’s raw body volume; if these differed by more than 150 mL, the BOD POD software instructed the technician to perform a third BV assessment and used the mean of the two closest values in subsequent computations. If no two measurements met the acceptance criteria, the entire trial was repeated.

Body fat percentage (%BF) was computed by the BOD POD’s software using the Siri equation, %BF = (4.95/BD − 4.5)·100%. Based on %BF, the software computed the subject’s fat-free mass (FFM) as well as her/his resting metabolic rate (RMR) [21].

### 2.3. Repeated Trials Protocols

To assess the test–retest reliability of single measurements, we analyzed the results obtained in the first two trials. In the case of repeated trials protocols, we analyzed the results obtained during the test protocol (composed of the first 3 trials) compared to those of the retest protocol (composed of the last 3 trials).

Three repeated trials protocols were compared: (i) the one proposed by Collins and McCarthy in their study of the precision of ADP [12] (hereafter the Collins protocol), (ii) the one devised by Tucker et al. [17] (the Tucker protocol), and (iii) the one proposed in the present work, which consists in taking the median of triplicate trials.

The Collins protocol requires to conduct at least two complete ADP trials. If they differ by at most 0.5% BF, the subject’s body composition variables are computed by taking the mean of the two readings; otherwise, a third trial is conducted and the results are computed by taking the mean of the closest pair of readings [12].

The Tucker protocol is similar to the Collins protocol, except for the largest acceptable difference between the first pair of %BF readings: it requires to conduct a third trial only if the first two differ by more than 1% BF [17].

The median protocol asks for triplicate trials regardless of the difference between the first two %BF readings, and the results of the body composition assessment are the ones that correspond to the median of the three %BF estimates.

### 2.4. Statistical Analysis

Bland–Altman (BA) plots [22,23,24] were used to characterize the repeatability of the measurements performed according to various protocols. The bias was computed as the mean value, $\bar{d}$, of the differences, $d_{i}$, between pairs of results (here, the index i labels subjects: i=1,2,…,n). The 95% limits of agreement were computed as $\bar{d}\pm 1.96SDd$, where SDddenotes the standard deviation of differences and the factor 1.96 is the two-sided *z*-score that corresponds to the 95% confidence level. We also represented the 95% confidence interval (95% CI) of the bias, $\bar{d}\pm t\cdot SDd/\sqrt{n}$, where t denotes the value at which the Student’s probability density function with n−1 degrees of freedom is equal to 0.05. For the upper limit of agreement (ULA), the 95% CI was computed as $\mathrm{ULA}\pm t\cdot SDd\cdot \sqrt{3/n}$, and a similar formula was used for the 95% CI of the lower limit of agreement (LLA) [23].

We applied the Shapiro–Wilk test to evaluate the normality of the distribution of the differences. The level of statistical significance was set to 0.05.

The TEM was obtained from Dahlberg’s formula [25]: $\mathrm{TEM}=\sqrt{\frac{1}{2n}\sum_{i=1}^{n}d_{i}^{2}}$.

ICC(2,1) was computed using the following relationship [18]:

$$\mathrm{ICC}(2,1)=\frac{MS_{S}-MS_{E}}{MS_{S}+(k-1)MS_{E}+k(MS_{T}-MS_{E})/n},$$

where k denotes the number of body composition tests being compared (here k=2), $MS_{s}$ is the subjects’ mean square, $MS_{E}$ is the error mean square, and $MS_{T}$ is the tests’ mean square. These mean square values were extracted from a two-way ANOVA table. The standard error of measurement was computed as $\mathrm{SEM}=SD\sqrt{1-\mathrm{ICC}(2,1)}$, where SD denotes the standard deviation of the test and retest results taken together (2n values). Finally, $\mathrm{MDC}=1.96\cdot \sqrt{2}\cdot \mathrm{SEM}$, where the factor $\sqrt{2}$ takes into account the variance of two measurements [26,27].

## 3. Results

### 3.1. Bland–Altman Analysis of Repeatability

**Figure 1:**
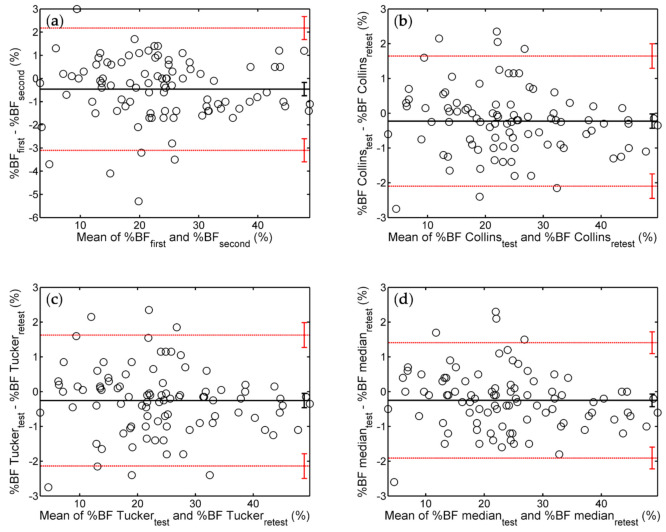
Bland–Altman (BA) analysis of the agreement between test and retest results of single ADP trials and three repeated trials protocols. Shown are plots of differences versus means of two assessments of %BF via (**a**) individual trials, (**b**) the Collins protocol, (**c**) the Tucker protocol, and (**d**) the median protocol. In each panel, the thick, solid, horizontal line depicts the bias (the mean value of the differences), whereas the red, thin, dotted, horizontal lines represent the 95% limits of agreement (bias ± standard deviation of the differences). Vertical error bars represent the 95% confidence intervals (95% CI) of the corresponding quantities.

In the context of reliability studies, BA plots represent the difference between two results obtained in measurements performed under identical conditions versus the mean of those results [22,23]. Figure 1 shows BA plots obtained for a pair of single trials (a), the Collins protocol [12] (b), the Tucker protocol [17] (c), and the median protocol proposed in this work (d). Each point of a BA plot corresponds to a pair of values obtained for the same person. The position of the point with respect to the horizontal axis reflects the mean adiposity of the subject.

In the absence of measurement errors, the two values would be identical, and all the points would be located on the horizontal axis; hence, the bias and the limits of agreement would be zero. Actual measurements are not error-free, so the points are scattered around the line that depicts the bias, with about 95% of them being located between LLA and ULA. The higher the test–retest reliability, the smaller the width of the 95% interval of agreement, ULA − LLA = 2 × (ULA − Bias).

The above interpretation of the limits of agreement is strictly valid only if the differences are normally distributed [22]. To evaluate this aspect, we applied the Shapiro–Wilk test and listed the corresponding *p*-values in the Supplementary Material, Table S1. Most of them were larger than 0.05, suggesting that the null hypothesis (which states that the differences come from a normal distribution with unspecified mean and variance) is true.

In the BA plots of Figure 1, the bias is slightly negative, and zero is marginally outside the corresponding 95% CI. Hence, compared to the test, the retest provides a higher estimate of the subject’s adiposity by about 0.3% BF. The 95% interval of agreement is widest for single measurements, indicating that repeated trials protocols are more reliable than individual ADP tests. Although it was more time-consuming, the Collins protocol did not exceed the Tucker protocol in reliability (compare panels b and c of Figure 1). The narrowest interval of agreement was observed for the median protocol (Figure 1, panel d).

**Figure 2:**
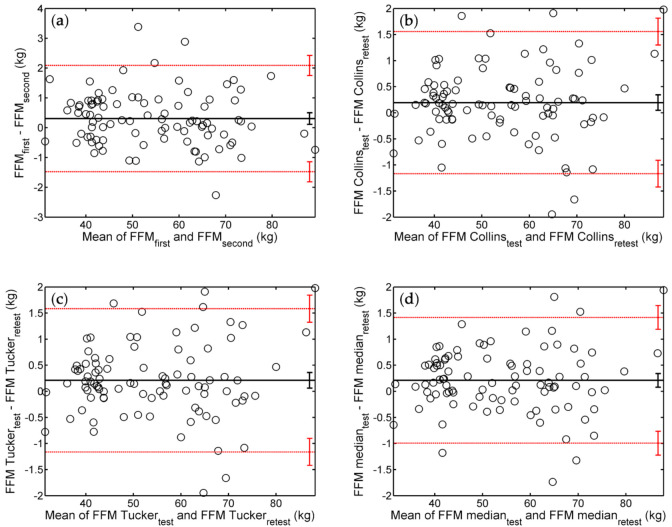
BA analysis of the reliability of FFM assessments via different protocols. The shown BA plots correspond to test–retest pairs of values obtained using (**a**) individual ADP trials, (**b**) the Collins protocol, (**c**) the Tucker protocol, and (**d**) the median protocol. Notations are explained in the caption of Figure 1.

Figure 2 shows the BA analysis of the agreement between successive FFM assessments by various protocols.

FFM measurements resulted in a bias of about 0.2 kg (i.e., on average, the retest provided higher FFM than the test). Again, the 95% interval of agreement was widest for individual tests, followed by the Collins and Tucker protocols (on roughly the same footing), and the median protocol.

Similar conclusions can be drawn from BA analyses performed for each sex, in part (see Supplementary Materials, Figures S1–S4). The corresponding values of the bias and ULA are listed in Table 2, along with their 95% CIs. When the analysis was performed separately for men and women, the 95% CIs were wider than those obtained for the entire study population because the sample size was half as large.

Table 3 summarizes the parameters of the BA analysis of the agreement between test and retest results for BV and RMR (see Supplementary Figures S5–S10).

According to Table 2 and Table 3, repeated trials resulted in a smaller bias than single measurements, and the associated 95% CI included zero in most of the cases. The bias was higher for women than for men, especially for BV and %BF assessments, as shown in the BA plots of Supplementary Figures S1, S2, S6 and S7.

The width of the 95% interval of agreement is provided by twice the difference between the ULA and the bias. A close scrutiny of Table 2 and Table 3 indicates that, for the entire sample, as well as for each sex in part, for all of the investigated body composition variables, the 95% interval of agreement was narrowest for the median protocol. Hence, according to the BA analysis, the median protocol is more reliable than the multiple trials protocols of References [12,17], which, in turn, are more reliable than single measurements.

For %BF, the width of the 95% interval of agreement for the median protocol was 3.36% BF for women and 3.3% BF for men (Table 2), indicating a slightly smaller precision for women than for men. For the other variables (FFM, BV, and RMR), the 95% interval of agreement was narrower in the case of women in the context of single measurements, as well as in the case of the investigated multiple trials protocols (Table 2 and Table 3).

In the BA plots of Figure 1 and Figure 2, and Supplementary Figures S1–S10, the markers are evenly distributed around the line of bias, indicating that, regardless of the applied protocol, the repeatability of ADP measurements does not depend on the subject’s body composition.

### 3.2. Absolute and Relative Measures of Reliability

Table 4 and Table 5 present statistical parameters that characterize the test–retest reliability of body composition assessments by different protocols.

The data presented in Table 4 and Table 5 reinforce the conclusion drawn from the BA analysis, that repeated trials protocols provide better reliability than single measurements. Among them, the median protocol proved to be the most reliable, whereas the protocol of Collins and McCarthy [12] was just marginally better than the one of Tucker et al. [17].

The reliability benefits of repeated trials protocols come with an increased workload. In this respect, the median protocol is the most demanding because it requires triplicate tests. In contrast, during the test procedure, the Collins protocol called for a third test for 62% of the participants, whereas the Tucker protocol required a third test for 42% of the subjects. Interestingly, these figures were lower (40% for Collins and 18% for Tucker) in the retest procedure, when the results of tests 4 and 5 were compared. Thus, if the time needed for calculations is not taken into account, the Collins protocol requires 2.4–2.6 times the effort of single trials, whereas the Tucker protocol is about 2.2–2.4-fold more time-consuming than single tests.

## 4. Discussion

In this paper, we evaluated the precision of body composition assessments by individual ADP trials and by three protocols based on duplicate or triplicate trials. Bland–Altman analysis as well as three absolute measures and one relative measure of reliability demonstrated that multiple trials offer better precision than single measurements. Hence, this work presents ways of pushing the precision of the BOD POD beyond the already good precision of the standard measurement procedure.

In the present study, the TEM of individual ADP trials was about 1% BF, in good agreement with the literature. Indeed, TEM values ranging from 0.55% to 1.28% BF were attained in investigations performed on different populations: Peeters found 0.55% BF for a sample of 21 young men [28], Peeters and Claessens obtained 0.57% BF for college-aged subjects (31 men and 31 women) [29], Collins and McCarthy reported 0.8% BF for a group of adults (45 men and 57 women) [12], Noreen and Lemon found 1.07% BF for a large, gender-balanced, heterogeneous sample of healthy adults (548 men and 432 women) [14], whereas Anderson obtained 1.28% BF for a group of 8 men and 16 women [30].

Despite the carefully designed measurement process, individual ADP trials can occasionally lead to rogue results, whose cause is as yet unknown. In a vast study of the BOD POD’s reliability [14], outliers were found for 32 of the 980 participants. In [14], an outlier was defined as a pair of trials that differed by at least 3% BF. In the present work, the first two trials differed by at least 3% BF for 6 subjects (3 men and 3 women) out of 92 participants, i.e., our percentage of outliers was about twice as large as that of [14], although the TEM was similar (see Table 4). When the outliers were eliminated from the database of [14], the TEM was reduced by 0.11% BF. In the present study, elimination of the outliers resulted in a decrement of 0.26% BF in the TEM and SEM, whereas the MDC decreased from 2.77% BF to 2.05% BF. Hence, our study reinforces the recommendation of Noreen and Lemon [14]: “Unless it can be determined how to eliminate these outliers, it is strongly advised that at least two repeated measures be performed to identify any outliers”. Gibson et al. also recommend to conduct duplicate measurements and report the mean values of the obtained body composition variables [31].

Besides spotting outliers, repeated trials protocols were proposed to boost the precision of ADP [17]. The present study compared three such protocols from the point of view of the test–retest reliability, and confirmed the hypothesis that repeated measures are more reliable than single ADP trials. Indeed, for all the body composition variables measured in this study, the width of the 95% interval of agreement, the TEM, the SEM, and the MDC were the largest, whereas ICC(2,1) was the smallest for single tests, reflecting the smallest (albeit still very good) test–retest reliability. Certain sets of differences between test and retest results deviated from the normal distribution (Supplementary Table S1). Despite these deviations, the 95% intervals of agreement were consistent with TEM, SEM, and MDC in ranking the test protocols according to their precision. Surprisingly, the more restrictive protocol due to Collins and McCarthy [12] did not perform better than the one proposed by Tucker et al. [17], perhaps because the largest acceptable difference between the first pair of trials in the Tucker protocol is roughly equal to the TEM of individual measurements. It seems reasonable to ask for a third measurement when the discrepancy between the first two exceeds the TEM (or SEM).

Thus, repeated trials provide reliability benefits, but the question arises whether they are worth the extra time and effort. When one seeks to track minute changes in body composition incurred during a dietary and/or lifestyle intervention, or to perform regular assessments in sports medicine [32], the answer is, probably, yes. Our study suggests that the most efficient repeated trials protocol available to date is the one by Tucker et al. [17] because, compared to single trials, it provides a 30% reduction in TEM, SEM, and MDC for about 2.4 times more effort. The median protocol, proposed in this work, proved to be the most reliable, but also the most time-consuming: it reduced the TEM, SEM, and MDC of %BF assessments by 38% at the cost of a 3-fold increase in testing time. Nevertheless, the median protocol has the advantage of comfortable data handling, and no calculations are required—one simply picks the results that correspond to the median of three consecutive measurements of %BF. Although the BOD POD is a highly reliable instrument, repeated trials protocols might be important in longitudinal studies that aim at detecting small changes in body composition over time.

The limitations of this study include the relatively small sample size and the focus on same-day measurements. Although our study group was too small to allow for stratification according to age or adiposity, it was sufficiently large to reveal the impact of gender on the measurement precision. The body composition tests analyzed in this study were conducted in close succession. Thus, the subject became used to the test procedure, and, therefore, the second triplet of values might have been less affected by errors related to the subject’s movement and/or breathing pattern. Further studies will be needed to clarify whether such learning effects indeed influence the precision of ADP.

## 5. Conclusions

Conducted on a heterogeneous, gender-balanced sample of healthy adults, this study evaluated the test–retest reliabilities of body composition tests conducted according to the protocol of Collins and McCarthy [9], the protocol of Tucker et al. [14], and the median protocol proposed in the present work.

The results of this study indicate that repeated trials protocols of body composition assessment by air displacement plethysmography are more reliable than the standard measurement procedure. Among them, the median protocol proved to be the most reliable. This conclusion was supported, for both genders, by Bland–Altman analysis and several statistical measures of test–retest reliability. Thus, evaluations of body volume, body fat percentage, fat-free mass, and resting metabolic rate can be performed with better precision using multiple measurements.

## Figures and Tables

**Table 1:** Subject characteristics, reported as mean ± SD and range of values [min., max.].

	All (*n* = 92)	Men (*n* = 46)	Women (*n* = 46)
Age (y)	30.4 ± 10.4 [20.0, 66.5]	29.6 ± 7.7 [20.3, 54.9]	31.2 ± 12.7 [20.0, 66.5]
Height (m)	1.71 ± 0.10 [1.49, 1.92]	1.79 ± 0.06 [1.69, 1.92]	1.63 ± 0.06 [1.49, 1.77]
BM ^1^ (kg)	71.6 ± 17.2 [38.0, 156.0]	80.6 ± 17.1 [57.5, 156.0]	62.6 ± 11.9 [38.0, 94.4]
BMI(kg/m^2^)	24.3 ± 4.6 [16.7, 45.1]	25.0 ± 4.6 [17.7, 45.1]	23.6 ± 4.6 [16.7, 33.7]
BV (L)	68.6 ± 17.1 [35.7, 155.4]	76.4 ± 17.7 [53.0, 155.4]	60.9 ± 12.5 [35.7, 94.9]
BSA (m^2^)	1.83 ± 0.24 [1.28, 2.72]	1.99 ± 0.20 [1.66, 2.72]	1.67 ± 0.15 [1.28, 2.06]
%BF ^2^ (%)	23.9 ± 10.8 [2.9, 49.6]	18.1 ± 8.8 [2.9, 42.9]	29.7 ± 9.5 [13.1, 49.6]
FFM ^2^ (kg)	54.1 ± 13.2 [31.0, 89.2]	65.0 ± 8.5 [50.6, 89.2]	43.3 ± 6.3 [31.0, 68.6]

^1^ Abbreviations: BM—body mass; BMI—body mass index; BV—body volume; BSA—body surface area; %BF—percent body fat; FFM—fat-free mass. ^2^ These quantities were determined using the repeated trials protocol of Tucker et al. [17] (see Section 2.3 for details).

**Table 2:** BA parameters of the repeatability of %BF and FFM assessments.

	Protocol	%BF (%)		FFM (kg)	
		Bias [95% CI]	ULA [95% CI]	Bias [95% CI]	ULA [95% CI]
All	Single	−0.46 [−0.75, −0.17]	2.18 [1.68, 2.68]	0.305 [0.111, 0.499]	2.089 [1.752, 2.425]
	Collins	−0.23 [−0.43, −0.03]	1.64 [1.29, 2.00]	0.194 [0.046, 0.343]	1.556 [1.299, 1.813]
	Tucker	−0.25 [−0.46, −0.05]	1.63 [1.27, 1.98]	0.211 [0.061, 0.360]	1.583 [1.324, 1.842]
	Median	−0.25 [−0.43, −0.07]	1.40 [1.09, 1.72]	0.210 [0.079, 0.341]	1.415 [1.188, 1.643]
Men	Single	−0.30 [−0.68, 0.07]	2.10 [1.45, 2.74]	0.237 [−0.050, 0.524]	2.093 [1.595, 2.591]
	Collins	−0.17 [−0.45, 0.12]	1.66 [1.17, 2.14]	0.175 [−0.059, 0.409]	1.683 [1.279, 2.088]
	Tucker	−0.20 [−0.50, 0.09]	1.70 [1.19, 2.21]	0.202 [−0.041, 0.444]	1.766 [1.346, 2.186]
	Median	−0.19 [−0.45, 0.06]	1.46 [1.01, 1.90]	0.199 [−0.014, 0.412]	1.574 [1.205, 1.943]
Women	Single	−0.62 [−1.06, −0.18]	2.23 [1.47, 2.99]	0.373 [0.107, 0.639]	2.092 [1.630, 2.553]
	Collins	−0.29 [−0.58, 0.01]	1.64 [1.12, 2.16]	0.227 [0.041, 0.413]	1.427 [1.105, 1.749]
	Tucker	−0.32 [−0.61, −0.03]	1.57 [1.06, 2.08]	0.220 [0.039, 0.401]	1.387 [1.074, 1.700]
	Median	−0.31 [−0.57, −0.05]	1.37 [0.92, 1.82]	0.222 [0.063, 0.380]	1.244 [0.970, 1.519]

Abbreviations: %BF—percent body fat, FFM—fat-free mass, ULA—upper limit of agreement.

**Table 3:** BA parameters for the reliability BV measurements and RMR estimates.

	Protocol	BV (L)		RMR (Kcal/Day)	
		Bias [95% CI]	ULA [95% CI]	Bias [95% CI]	ULA [95% CI]
All	Single	−0.051 [−0.091, −0.012]	0.312 [0.244, 0.381]	6.728 [2.505, 10.952]	45.489 [38.173, 52.805]
	Collins	−0.014 [−0.043, 0.016]	0.259 [0.207, 0.310]	4.136 [0.959, 7.313]	33.290 [27.787, 38.792]
	Tucker	−0.020 [−0.050, 0.011]	0.260 [0.206, 0.312]	4.739 [1.478, 8]	34.663 [29.015, 40.311]
	Median	−0.019 [−0.045, 0.007]	0.224 [0.178, 0.269]	4.598 [1.750, 7.446]	30.735 [25.802, 35.668]
Men	Single	−0.036 [−0.094, 0.023]	0.341 [0.240, 0.442]	5.261 [−0.991, 11.513]	45.619 [34.790, 56.447]
	Collins	−0.005 [−0.053, 0.042]	0.302 [0.219, 0.384]	3.837 [−1.228, 8.902]	36.532 [27.760, 45.304]
	Tucker	−0.012 [−0.061, 0.037]	0.304 [0.219, 0.388]	4.413 [−0.845, 9.671]	38.358 [29.25, 47.465]
	Median	−0.014 [−0.057, 0.029]	0.263 [0.189, 0.337]	4.326 [−0.275, 8.927]	34.025 [26.057, 41.994]
Women	Single	−0.067 [−0.121, −0.012]	0.284 [0.190, 0.379]	8.196 [2.415, 13.976]	45.512 [35.499, 55.524]
	Collins	−0.022 [−0.059, 0.016]	0.221 [0.156, 0.286]	4.728 [0.638, 8.819]	31.133 [24.048, 38.217]
	Tucker	−0.025 [−0.062, 0.011]	0.212 [0.149, 0.276]	5.065 [1.093, 9.038]	30.710 [23.829, 37.591]
	Median	−0.023 [−0.055, 0.008]	0.181 [0.126, 0.236]	4.870 [1.410, 8.329]	27.204 [21.212, 33.197]

Abbreviations: BV—body volume, RMR—resting metabolic rate, ULA—upper limit of agreement.

**Table 4:** Statistical measures of test–retest reliability of %BF and FFM measurements.

	Protocol	%BF (%)				FFM (kg)			
		TEM ^1^	SEM	MDC	ICC(2,1) ^2^	TEM	SEM	MDC	ICC(2,1)
All	Single	1.00	1.00	2.77	0.9914	0.675	0.673	1.867	0.9974
	Collins	0.69	0.69	1.91	0.9960	0.507	0.506	1.403	0.9985
	Tucker	0.70	0.70	1.93	0.9959	0.515	0.513	1.422	0.9985
	Median	0.62	0.62	1.72	0.9967	0.457	0.456	1.264	0.9988
Men	Single	0.88	0.88	2.44	0.9898	0.683	0.679	1.883	0.9934
	Collins	0.66	0.66	1.82	0.9944	0.552	0.549	1.522	0.9957
	Tucker	0.69	0.69	1.91	0.9938	0.576	0.573	1.588	0.9953
	Median	0.60	0.60	1.67	0.9953	0.510	0.508	1.407	0.9963
Women	Single	1.11	1.10	3.05	0.9866	0.668	0.664	1.840	0.9885
	Collins	0.72	0.71	1.98	0.9944	0.457	0.455	1.261	0.9948
	Tucker	0.71	0.71	1.96	0.9945	0.444	0.442	1.225	0.9951
	Median	0.64	0.63	1.76	0.9956	0.397	0.395	1.095	0.9961

Abbreviations: %BF —percent body fat, FFM—fat-free mass, TEM—technical error of measurement, SEM—standard error of measurement, MDC—minimal detectable change, ICC—intraclass correlation coefficient. ^1^ TEM, SEM, and MDC are expressed in the same units as the corresponding body composition variable (% for %BF and kg for FFM); the smaller they are, the higher the reliability. ^2^ ICC(2,1) is dimensionless and ranges from 0 to 1—the higher the better.

**Table 5:** Statistical parameters of the reliability of various protocols for BV measurements and RMR estimates provided by the BOD POD software relying on measured fat mass and fat-free mass [21].

	Protocol	BV (L)				RMR (kcal/day)			
		TEM	SEM	MDC	ICC(2,1)	TEM	SEM	MDC	ICC(2,1)
All	Single	0.135	0.135	0.374	0.9999	14.7	14.7	40.6	0.9982
	Collins	0.098	0.098	0.271	1.0000	10.9	10.8	30.0	0.9990
	Tucker	0.101	0.101	0.280	1.0000	11.2	11.2	31.1	0.9989
	Median	0.088	0.088	0.243	1.0000	9.9	9.9	27.4	0.9992
Men	Single	0.137	0.136	0.377	0.9999	14.9	14.8	41.0	0.9962
	Collins	0.110	0.109	0.302	1.0000	12.0	11.9	33.0	0.9975
	Tucker	0.113	0.112	0.311	1.0000	12.5	12.4	34.5	0.9973
	Median	0.099	0.099	0.274	1.0000	11.0	11.0	30.4	0.9979
Women	Single	0.134	0.133	0.369	0.9999	14.5	14.4	40.0	0.9926
	Collins	0.088	0.088	0.243	1.0000	10.0	9.9	27.6	0.9966
	Tucker	0.087	0.086	0.239	1.0000	9.8	9.8	27.1	0.9967
	Median	0.075	0.074	0.206	1.0000	8.7	8.6	23.9	0.9974

Abbreviations are explained in the footer of Table 4.

## Data Availability

The data generated during this study have been anonymized and included in the Supplementary Materials.

## Supplementary Materials

The following are available online at https://www.mdpi.com/article/10.3390/ijerph182010693/s1, Figure S1: Bland–Altman (BA) analysis of the reliability of various protocols for %BF assessments of men, Figure S2: BA analysis of the reliability of repeat measures protocols for %BF evaluation of women, Figure S3: BA analysis of the reliability of FFM assessments of men, Figure S4: BA analysis of the reliability of FFM measurements of women, Figure S5: BA plots illustrating the reliability of BV measurements in the entire sample, Figure S6: BA analysis of the reliability of BV assessments of men, Figure S7: BA analysis of the reliability of BV measurements of women, Figure S8: BA analysis of the reliability of RMR estimates of all the subjects involved in this study, Figure S9: BA plots illustrating the repeatability of RMR assessments of men, Figure S10: BA analysis of the test–retest reliability of RMR estimations in the case of women, Table S1: *p*-values of the Shapiro–Wilk test to determine whether the differences between the test and retest results are normally distributed. Data S1: Anonymized data file.
