# Supplementary material for: Reliability of Repeated Trials Protocols for Body Composition Assessment by Air Displacement Plethysmography
Source: Int J Environ Res Public Health. 2021 Oct 12;18(20):10693. doi: 10.3390/ijerph182010693 (PMC8535236; doi:10.3390/ijerph182010693)
Supplement: Supplementary file 1 [file ijerph-18-10693-s001.zip › ijerph-1346684-supplementary/ADPreptrials_Supplementary_Materials_R1.pdf]

SUPPLEMENTARY MATERIALS

for the article

Reliability of repeated trials protocols for body composition assessment by air displacement plethysmography

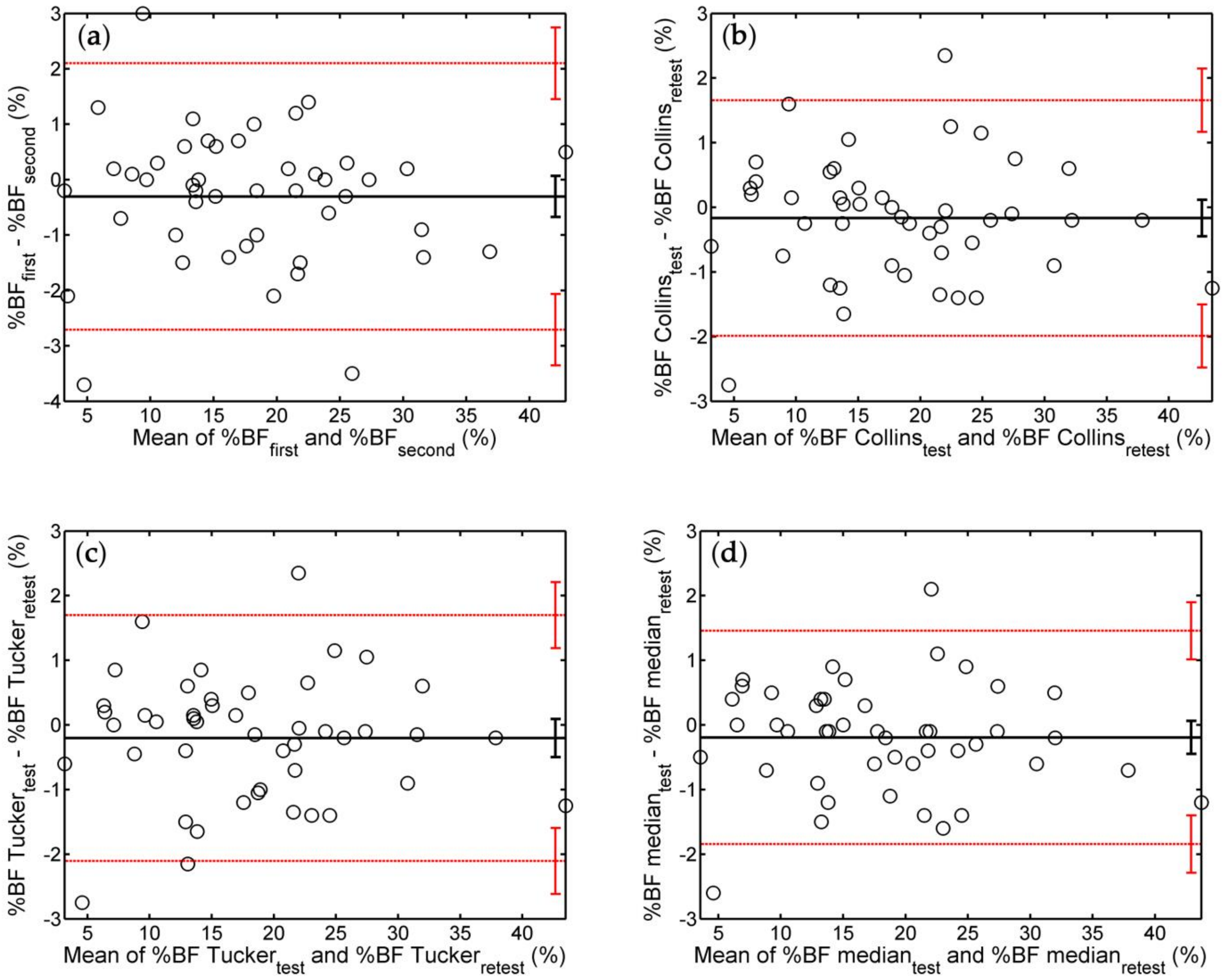

Figure S1. Bland-Altman (BA) analysis of the reliability of various protocols for %BF assessments of men. Shown are plots of differences vs. means of test-retest pairs of values from (**a**) individual ADP trials (**b**) the Collins protocol, (**c**) the Tucker protocol, and (**d**) the median protocol. (See the caption of Figure 1 for notations).

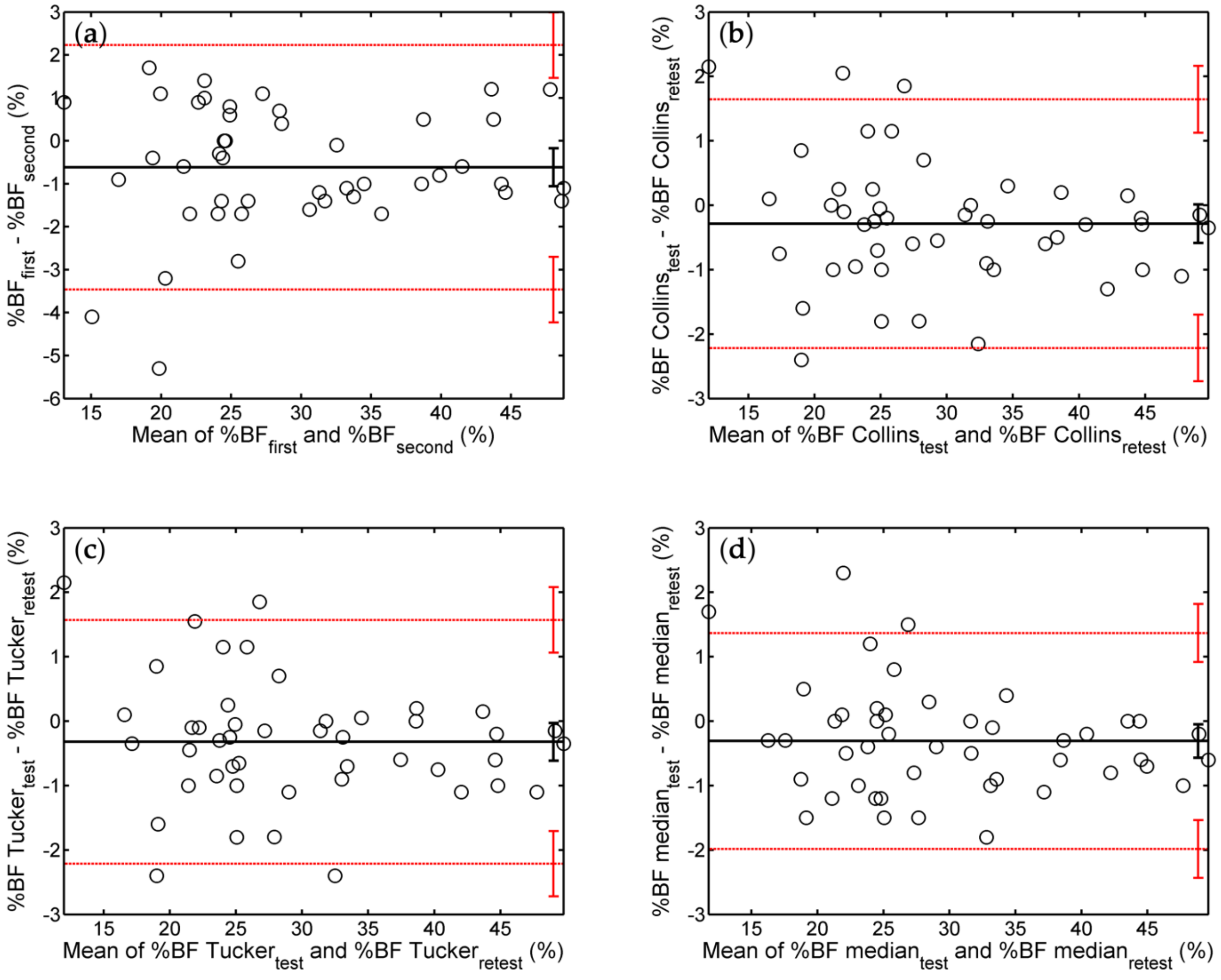

Figure S2. BA analysis of the reliability of repeat measures protocols for %BF evaluation of women. Plotted are differences vs. means of test and retest results given by 4 different protocols: (**a**) individual tests (**b**) Collins, (**c**) Tucker, and (**d**) median.

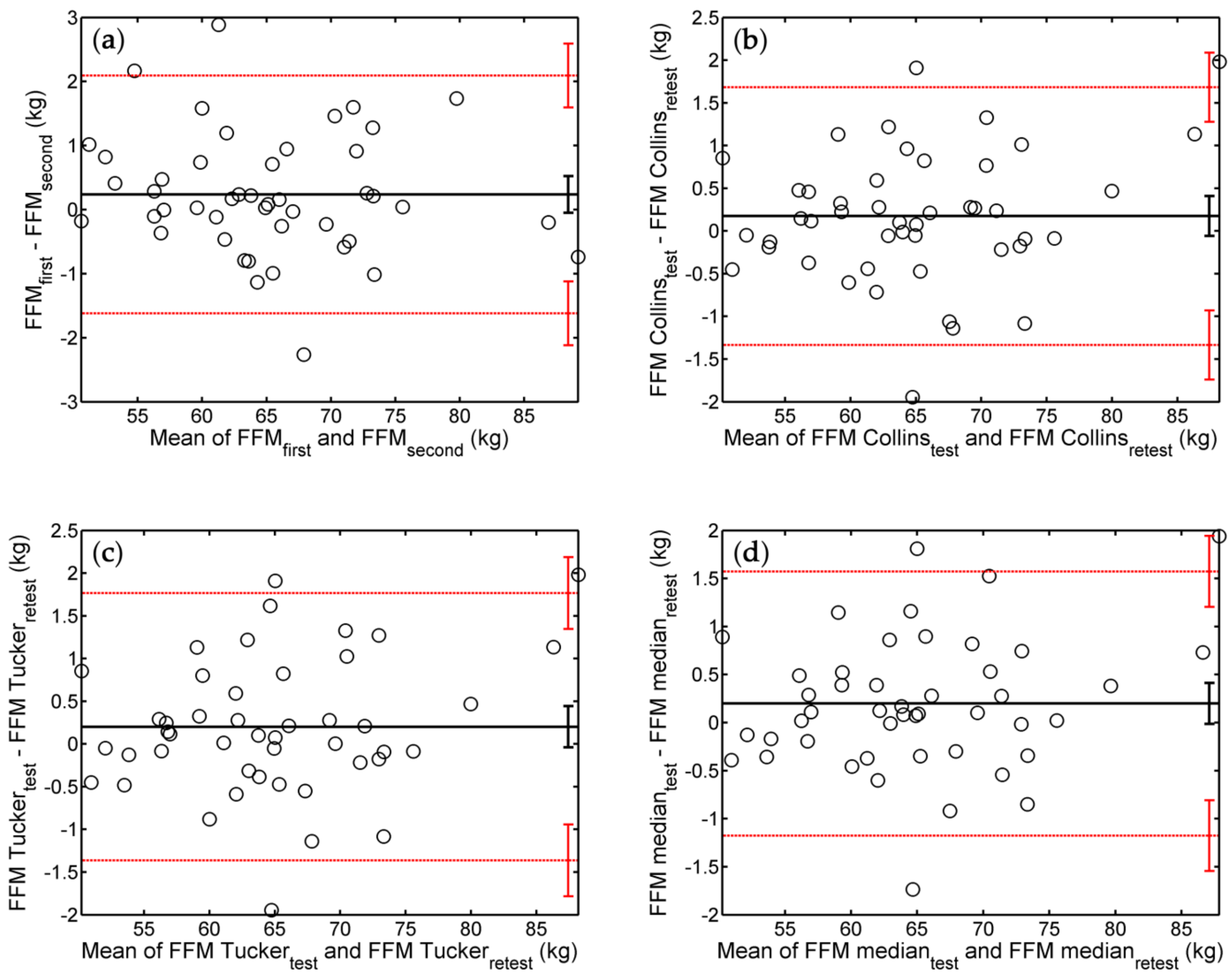

Figure S3. BA analysis of the reliability of FFM assessments of men. Shown are plots of differences vs. means of test and retest values inferred from 4 different protocols: (**a**) individual tests (**b**) Collins, (**c**) Tucker, and (**d**) median.

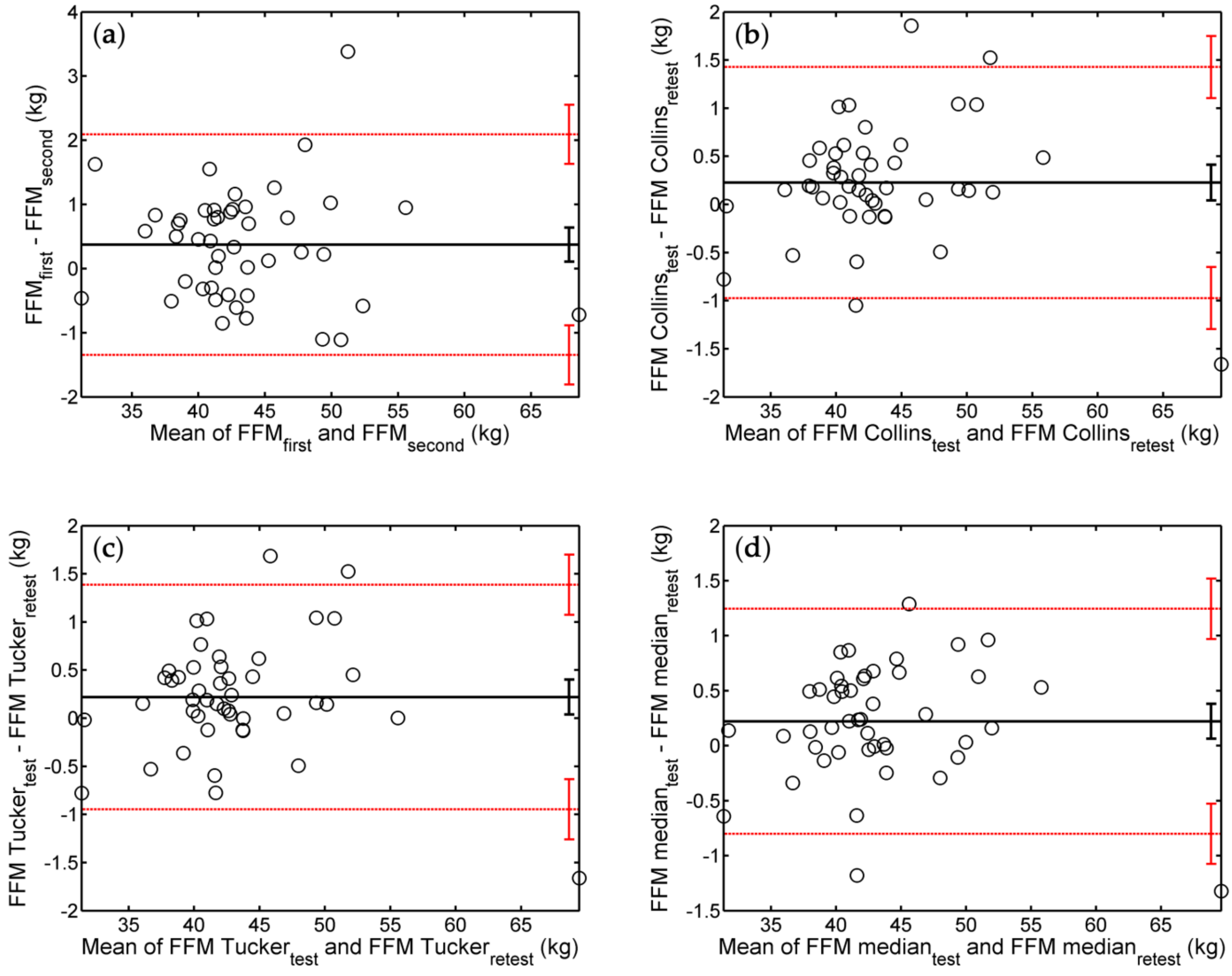

Figure S4. BA analysis of the reliability of FFM measurements of women. Plotted are differences vs. means of test-retest pairs of values given by 4 different protocols: (**a**) individual tests (**b**) Collins, (**c**) Tucker, and (**d**) median.

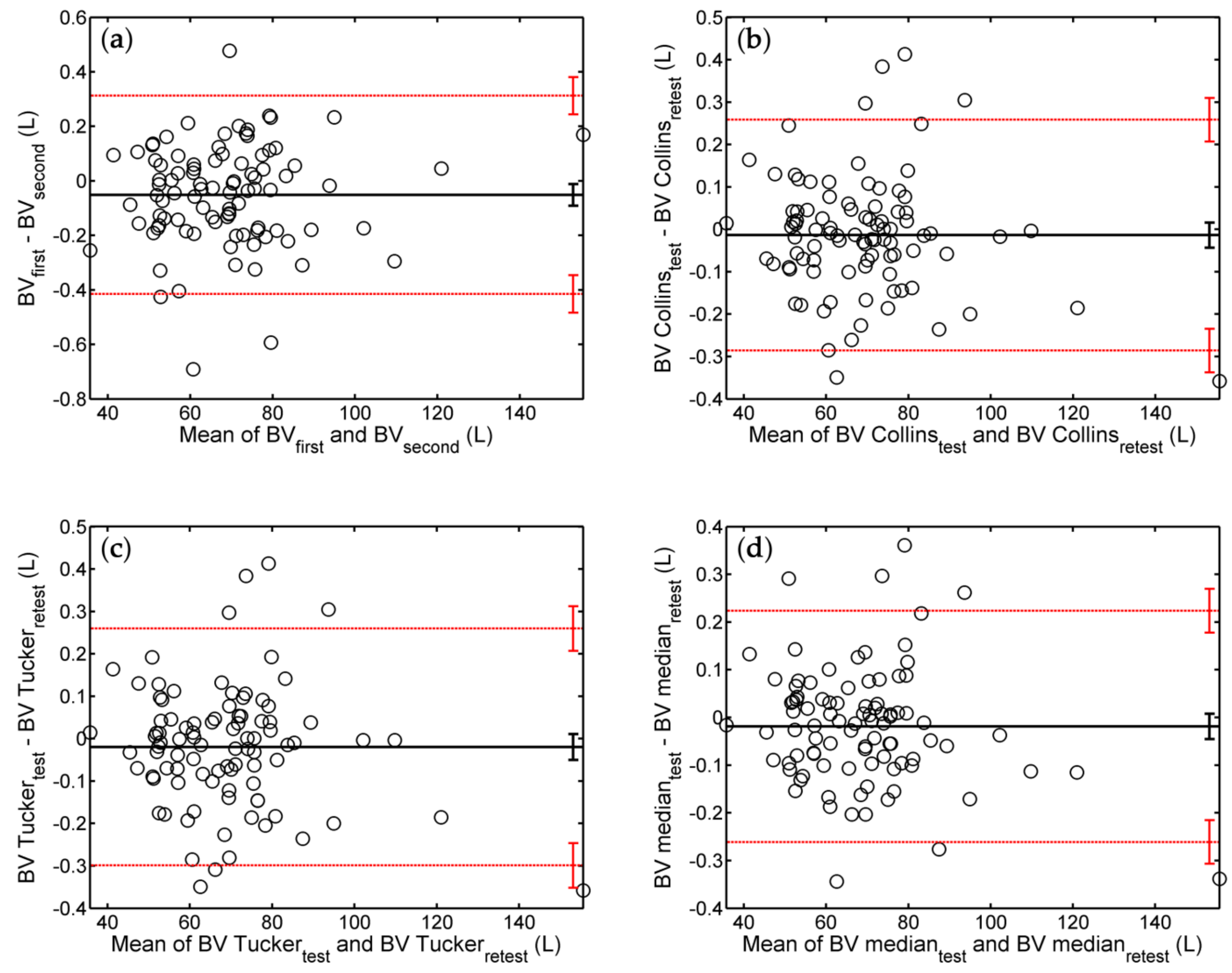

Figure S5. BA plots illustrating the reliability of BV measurements in the entire sample. The plots represent differences vs. means of test-retest pairs of values obtained via the following protocols: (**a**) individual trials (**b**) Collins, (**c**) Tucker, and (**d**) median.

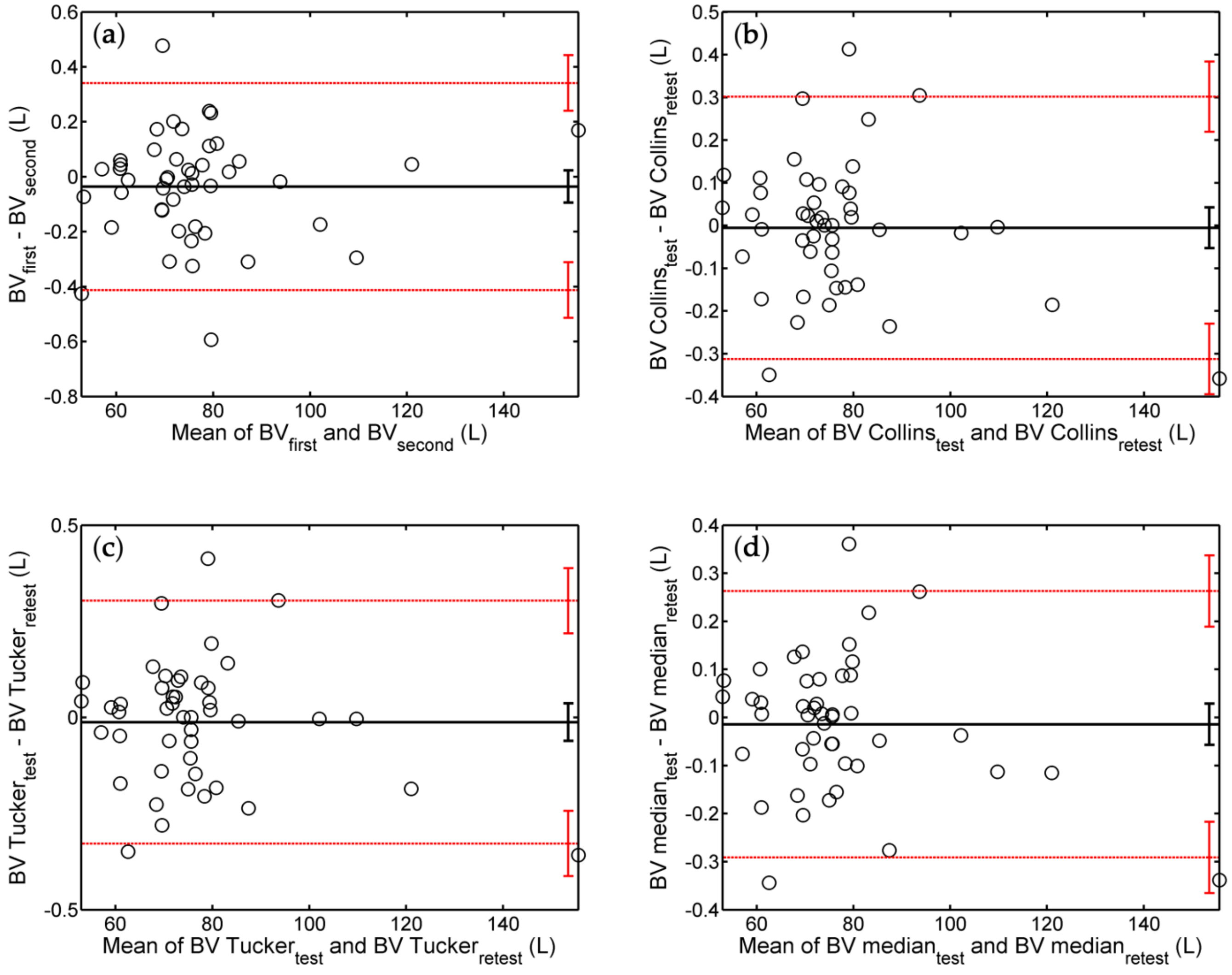

Figure S6. BA analysis of the reliability of BV assessments of men. The 4 panels represent differences vs. means of test and retest values obtained via the following protocols: (**a**) individual trials (**b**) Collins, (**c**) Tucker, and (**d**) median.

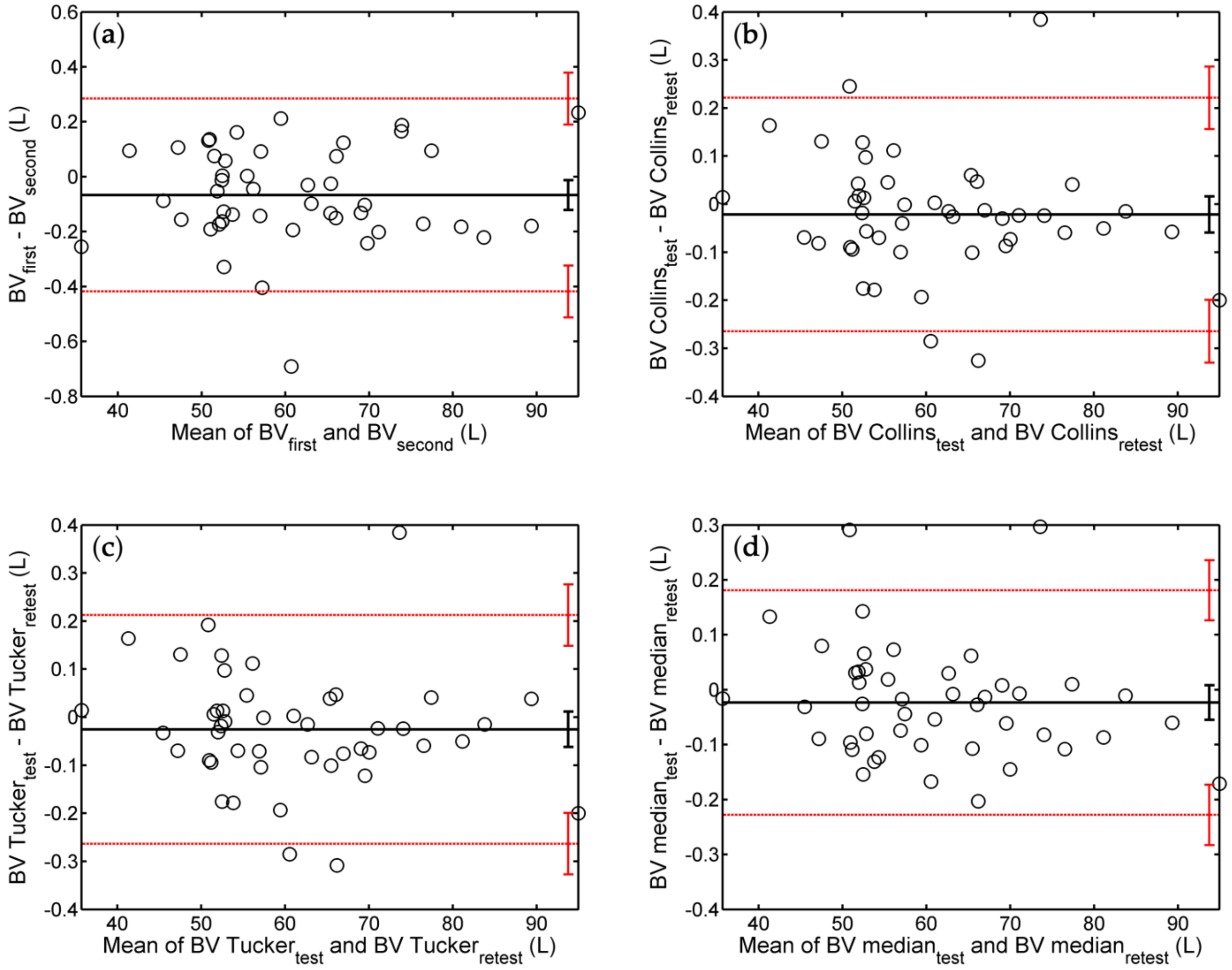

Figure S7. BA analysis of the reliability of BV measurements of women. The shown plots represent differences vs. means of test and retest values given by the following protocols: (**a**) individual trials (**b**) Collins, (**c**) Tucker, and (**d**) median.

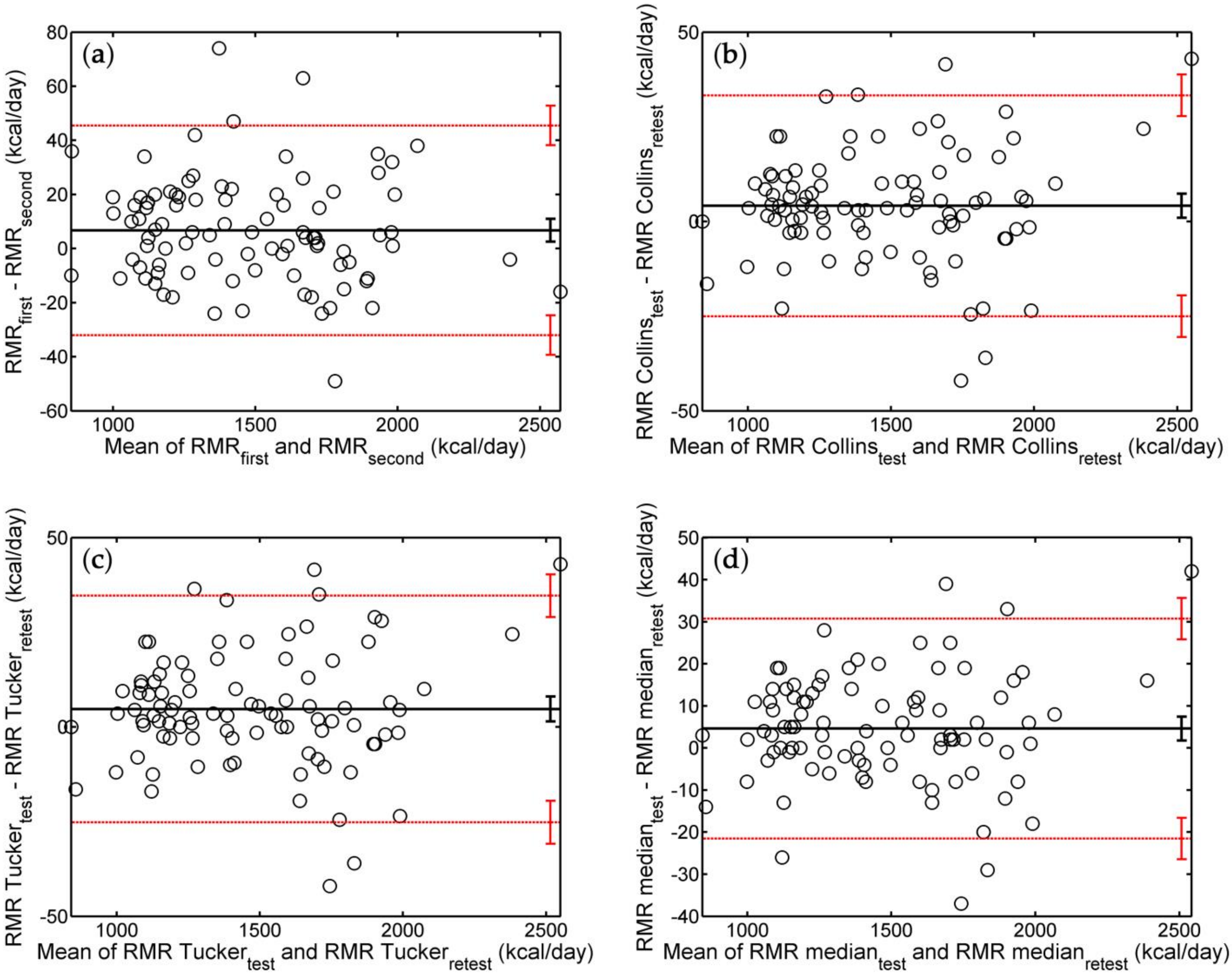

Figure S8. BA analysis of the reliability of RMR estimates of all the subjects involved in this study. The shown plots represent differences vs. means of test-retest pairs of values obtained via 4 different protocols: (**a**) individual trials (**b**) Collins, (**c**) Tucker, and (**d**) median.

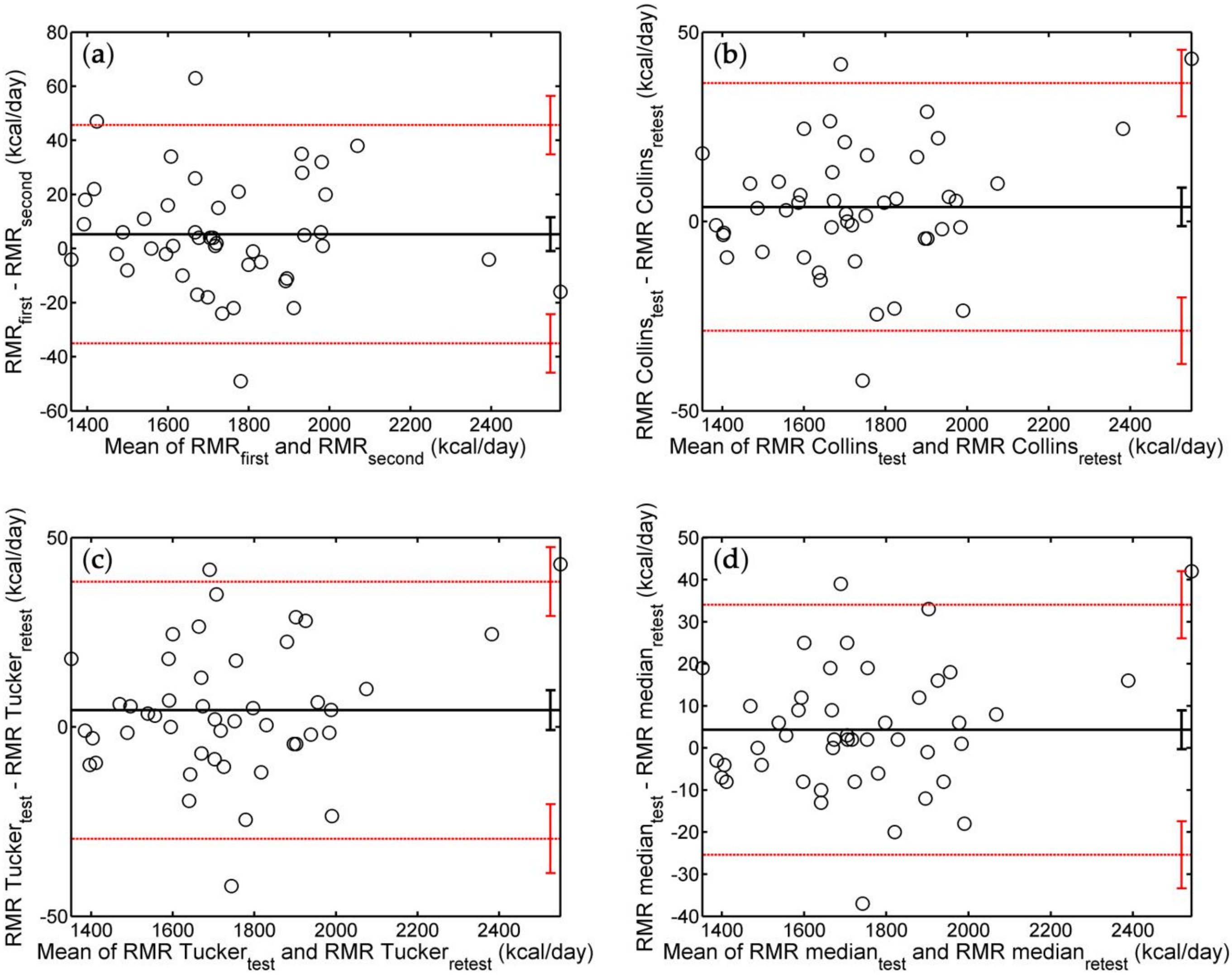

Figure S9. BA plots illustrating the repeatability of RMR assessments of men. Shown are plots of differences vs. means of the RMR values obtained during the test and retest procedure according to the following protocols: (**a**) individual tests (**b**) Collins, (**c**) Tucker, and (**d**) median.

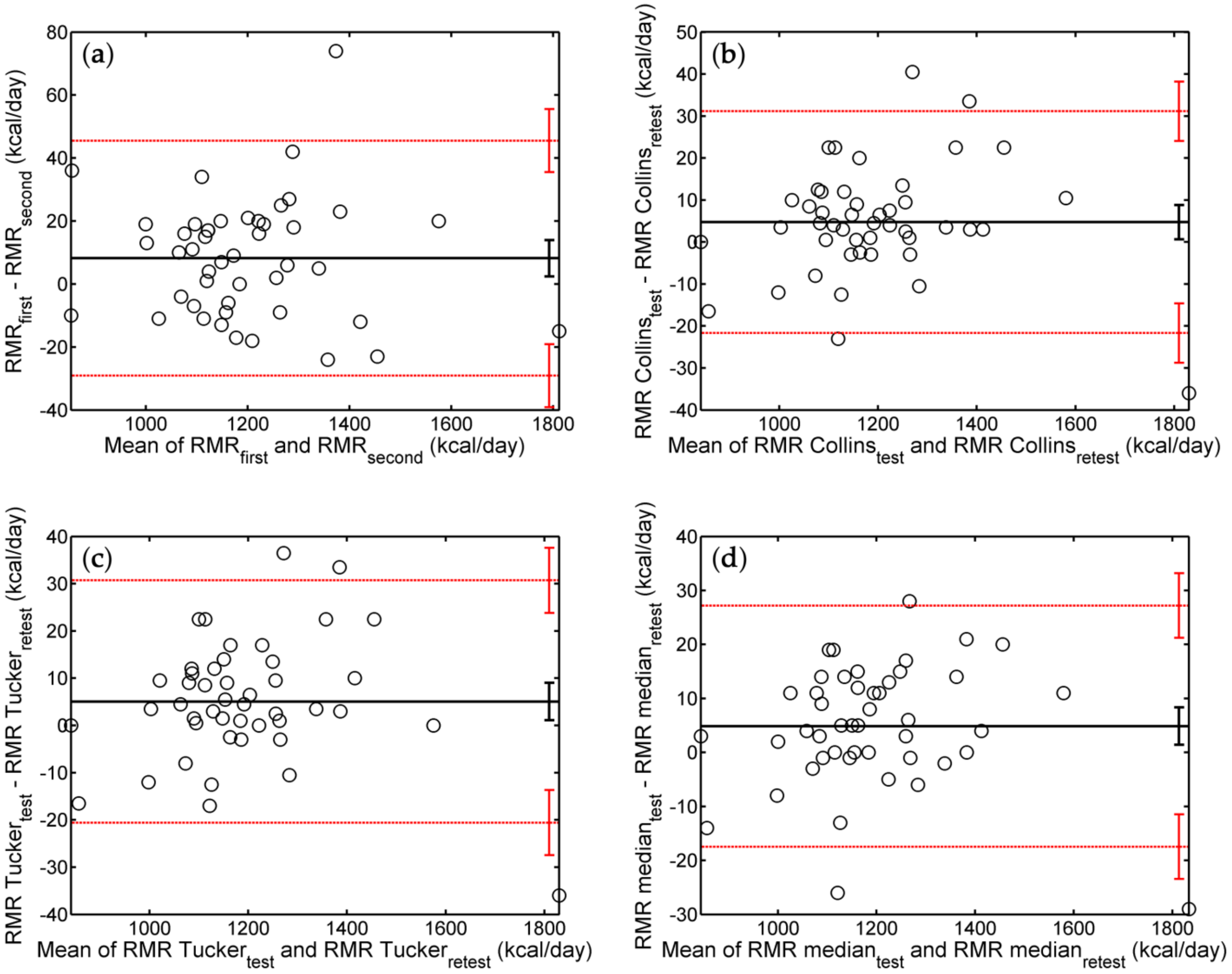

Figure S10. BA analysis of the test-retest reliability of RMR estimations in the case of women. The shown plots represent differences vs. means of the RMR values obtained during test and retest by applying 4 different protocols: (**a**) individual trials (**b**) Collins, (**c**) Tucker, and (**d**) median.

Table S1. *P*-values of the Shapiro-Wilk test to determine whether the differences between the test and retest results are normally distributed. The null hypothesis of this test states that the data comes from a normal distribution with unspecified mean and variance. *P*-values smaller than 0.05 question the validity of the null hypothesis, indicating that the data deviates from a normal distribution.

		**%BF (%)**	**FFM (kg)**	**BV (L)**	**RMR (kcal/day)**
**All**	Single	0.004	0.107	0.057	0.090
	Collins	0.047	0.023	0.007	0.024
	Tucker	0.359	0.098	0.063	0.108
	Median	0.205	0.215	0.064	0.226
**Men**	Single	0.096	0.433	0.296	0.452
	Collins	0.836	0.718	0.378	0.741
	Tucker	0.702	0.398	0.282	0.399
	Median	0.531	0.429	0.646	0.444
**Women**	Single	0.010	0.036	0.019	0.030
	Collins	0.020	0.025	0.012	0.025
	Tucker	0.151	0.154	0.110	0.167
	Median	0.025	0.056	0.007	0.052
